# Trendelenburg maneuver predicts fluid responsiveness in patients on veno-arterial extracorporeal membrane oxygenation

**DOI:** 10.1186/s13613-021-00811-x

**Published:** 2021-01-26

**Authors:** Jing-chao Luo, Ying Su, Li-li Dong, Jun-yi Hou, Xin Li, Ying Zhang, Guo-guang Ma, Ji-li Zheng, Guang-wei Hao, Huan Wang, Yi-jie Zhang, Zhe Luo, Guo-wei Tu

**Affiliations:** 1 Department of Critical Care Medicine, Zhongshan Hospital, Fudan University, Shanghai, China; 2 Department of Echocardiography, Zhongshan Hospital, Fudan University, Shanghai, China; 3 Shanghai Institute of Medical Imaging, Zhongshan Hospital, Fudan University, Shanghai, China; 4 Department of Cardiovascular Surgery, Zhongshan Hospital, Fudan University, Shanghai, China; 5 Department of Nursing, Zhongshan Hospital, Fudan University, Shanghai, China; 6 Department of Critical Care Medicine, Xiamen Branch, Zhongshan Hospital, Fudan University, Xiamen, China

**Keywords:** Fluid responsiveness, Trendelenburg maneuver, Veno-arterial extracorporeal membrane oxygenation

## Abstract

**Background:**

Evaluation of fluid responsiveness during veno-arterial extracorporeal membrane oxygenation (VA-ECMO) support is crucial. The aim of this study was to investigate whether changes in left ventricular outflow tract velocity–time integral (ΔVTI), induced by a Trendelenburg maneuver, could predict fluid responsiveness during VA-ECMO.

**Methods:**

This prospective study was conducted in patients with VA-ECMO support. The protocol included four sequential steps: (1) baseline-1, a supine position with a 15° upward bed angulation; (2) Trendelenburg maneuver, 15° downward bed angulation; (3) baseline-2, the same position as baseline-1, and (4) fluid challenge, administration of 500 mL gelatin over 15 min without postural change. Hemodynamic parameters were recorded at each step. Fluid responsiveness was defined as ΔVTI of 15% or more, after volume expansion.

**Results:**

From June 2018 to December 2019, 22 patients with VA-ECMO were included, and a total of 39 measurements were performed. Of these, 22 measurements (56%) met fluid responsiveness. The *R*^2^ of the linear regression was 0.76, between ΔVTIs induced by Trendelenburg maneuver and the fluid challenge. The area under the receiver operating characteristic curve of ΔVTI induced by Trendelenburg maneuver to predict fluid responsiveness was 0.93 [95% confidence interval (CI) 0.81–0.98], with a sensitivity of 82% (95% CI 60–95%), and specificity of 88% (95% CI 64–99%), at a best threshold of 10% (95% CI 6–12%).

**Conclusions:**

Changes in VTI induced by the Trendelenburg maneuver could effectively predict fluid responsiveness in VA-ECMO patients.

*Trial registration* ClinicalTrials.gov, NCT 03553459 (the TEMPLE study). Registered on May 30, 2018

## Background

Veno-arterial extracorporeal membrane oxygenation (VA-ECMO) is a rescue therapy for patients with refractory cardiogenic shock [1, 2]. During VA-ECMO support, hypotension may frequently occur due to deteriorated cardiac function, vasoplegia, or hypovolemia [3, 4]. Volume expansion is a common means to correct hypotension and improve systemic perfusion, but inappropriate fluid therapy is associated with adverse outcomes [5]. Prediction of fluid responsiveness before fluid resuscitation could achieve a lower fluid balance, reduce the risk of renal and respiratory failure, and improve outcomes for critically ill patients [6].

Several methods are currently available to evaluate fluid responsiveness in critically ill patients [7–9]. Mechanical ventilation generates a cyclical change in intra-thoracic pressure and venous return. Based on this interaction, stroke volume variation (SVV) and pulse pressure variation (PPV) are often used as dynamic parameters to predict fluid responsiveness [8]. However, VA-ECMO patients frequently present with low tidal volume, cardiac arrhythmia and especially pulselessness, thus making these parameters less reliable [8, 10, 11]. Respiratory variations in the inferior vena cava diameter (ΔIVCD) have previously been used as accurate predictors for fluid responsiveness in mechanically ventilated patients [12]. However, drainage cannulation placed in the inferior vena cava impedes the application of ΔIVCD. Simulation of a revisable “autotransfusion” is another feasible approach to assess fluid responsiveness. Passive leg raising (PLR) test transiently increases venous return by postural maneuver [8]. Unfortunately, the risk of cannula distortion or displacement precluded the application of this maneuver to VA-ECMO patients. Overall, the physiological features of VA-ECMO patients restrict the use of conventional methods to assess fluid responsiveness [11].

The Trendelenburg maneuver is a “self” volumetric loading maneuver [13], and has demonstrated good accuracy in predicting responsiveness for acute respiratory distress syndrome [14] and surgical patients [15], and can be performed for the majority of VA-ECMO patients. Transthoracic echocardiography (TTE) is now routinely used to evaluate cardiac function recovery, and allows the measurement of stroke volume and cardiac output in VA-ECMO patients [16]. Thus, the combination of the Trendelenburg maneuver and TTE facilitates the evaluation of fluid responsiveness during VA-ECMO support.

This study was designed to investigate whether a change in velocity–time integral (ΔVTI) measured by TTE induced by the Trendelenburg maneuver, could predict fluid responsiveness in patients on VA-ECMO.

## Methods

### Patients

This study was approved by the Institutional Review Board (Zhongshan Hospital, Fudan University, Shanghai, China: Number B2018-074), and conducted in a 40-bed cardiac surgical intensive care unit (ICU). During the study period (June 2018–December 2019), 22 postoperative patients were prospectively enrolled after informed consent was received from the patient’s next of kin. We included ventilated patients with relatively low VA-ECMO pump flow (2–3 L/min) for whom the decision to perform volume expansion was made by the attending physician (hypotension, hypoperfusion [oliguria or skin mottling], or attempt to reduce vasopressor dose [17, 18]). Exclusion criteria were patients < 18 years old, pregnant, pulselessness (pulse pressure (PP) < 15 mmHg [19]), contraindication to the Trendelenburg position (cerebral edema, intra-abdominal hypertension and gastric retention), or unsatisfactory cardiac echogenicity (an inability to correctly align the Doppler beam to generate reliable VTI measurements at the left ventricular outflow tract [LVOT]). Patients with evidence of significant hypovolemia, such as kicking drainage cannula [20] (suggesting a transient venous or atrial collapse) and persistent hemorrhage, were also excluded.

### Protocol

**Fig. 1:**
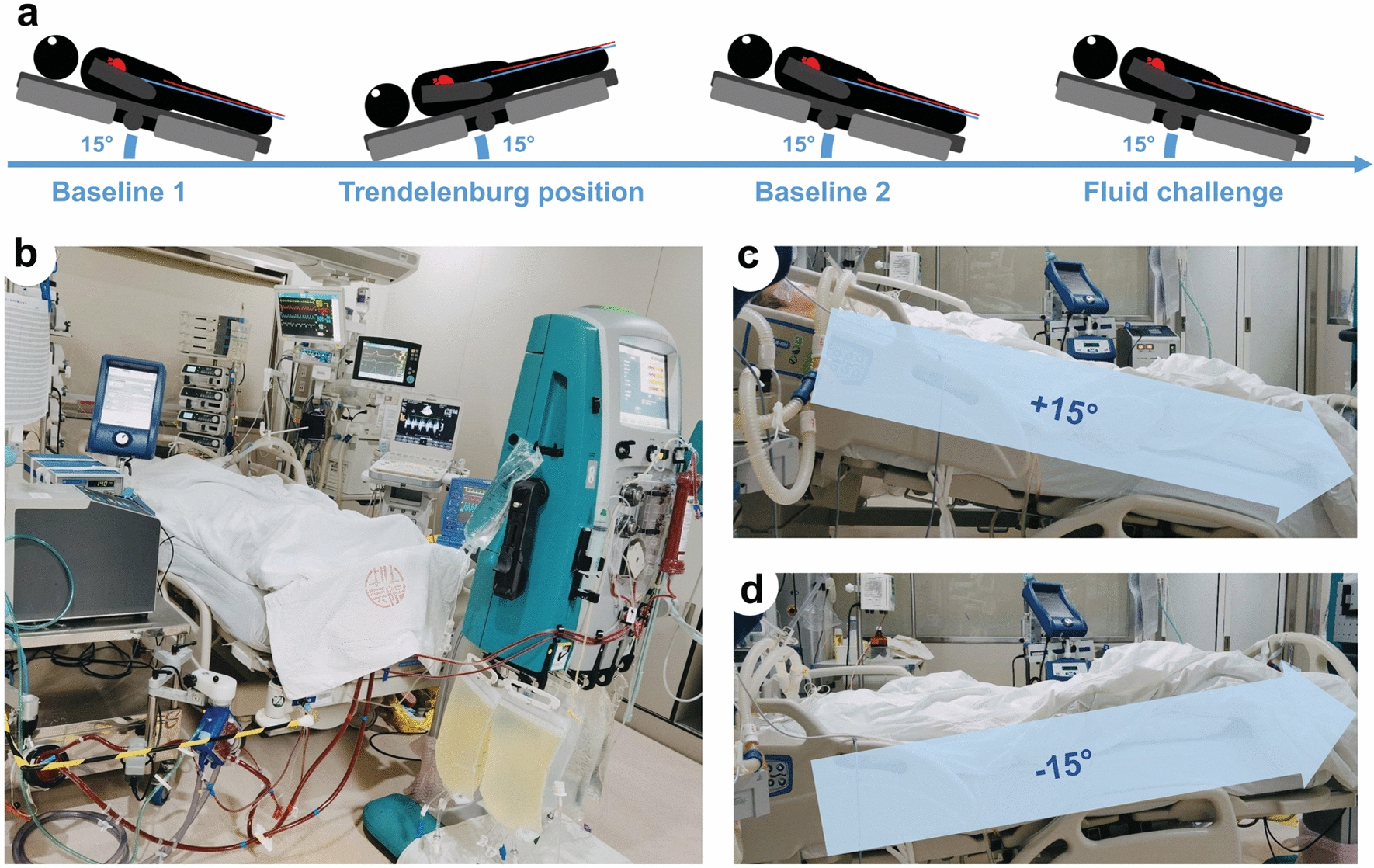
The study protocols. **a** An outline of each step; **b** a clinical scenery of VA-ECMO patient; **c** the 15° upward bed angulation for measurements at baselines and fluid challenge**. d** The 15° downward bed angulation for measurements in the Trendelenburg position

Throughout the study, patients were sedated with a combination of remifentanil and midazolam, with the aim of achieving a Richmond Agitation–Sedation Scale [21] of − 5. The protocol included four sequential steps (Fig. 1):

1. Baseline-1: a supine position with a 15° upward bed angulation,
2. Trendelenburg maneuver: 15° downward bed angulation [22],
3. Baseline-2: recover to the same position as baseline-1, and
4. Fluid challenge: administration of 500 mL gelatin over 15 min without postural change.

After 1 min stabilization for each step, VTI, heart rate (HR), systolic blood pressure (SBP), diastolic blood pressure (DBP), central venous pressure (CVP) and pulse pressure (PP) were recorded. In our center, the tip of the drainage cannula was placed in the right atrium to guarantee adequate blood collection [11]. The pressure transducer was fixed at the level of the patient’s right atrium, which was located at the intersection of the mid-axillary line and the fourth interspace. During the study, the pump was maintained at the same rotation speed. Other therapies such as sedation, vasopressors and ventilation also remained unchanged. To acquire as much data as feasible, some patients underwent the test more than once (on different days) if required, therefore 39 measurements were conducted and recorded.

### VTI measurements

Transthoracic echocardiography was performed on a CX50 instrument (Philips Healthcare, Eindhoven, The Netherlands) by the same experienced operator (LL Dong) who was blinded to patient clinical data. VTI was measured at the level of the LVOT, using the 5-chamber apical view. Five consecutive measurements were recorded to calculate a mean VTI value. For TTE measurement, some indices were defined to estimate the reproducibility [17, 23]: (1) coefficient of variation (CV) = standard deviation (SD)/mean; (2) coefficient of error (CE) = CV/√*n* (the number of replicate TTE); (3) precision = 2 × CE; (4) least significant change (LSC) = CE × 1.96 × √2, indicting the minimal observed change value can be considered as real. These assessments were performed in eight patients at baseline position by obtaining twice by the same operator (LL Dong; intra-observer reproducibility) and a second operator (Y Liu; inter-observer reproducibility). The CV was 4% for intra-observer variability, and 5% for inter-observer variability. The intra-observer LSC was 8% for VTI measurements.

### Data collection

Upon patient inclusion, demographic information, surgical procedures, Acute Physiologic and Chronic Health Evaluation (APACHE) II scores, left ventricular ejection fraction (LVEF), laboratory examinations and blood gas analyses were collected. Supportive therapies included VA-ECMO and mechanical ventilation (MV) indices, vasoactive drug doses (transferred to equivalent inotropic scores) [24], and the concomitant use of intra-aortic balloon pumps (IABP), renal replacement therapy (RRT) or inspired nitric oxide (iNO). We recorded all data related to these parameters. All study patients were followed up until hospital discharge or death, to record clinical outcomes, such as length of MV, tracheotomy rate, length of ICU stay, length of hospital stay and hospital mortality.

### Statistical analysis

Sample size estimation was performed using PASS software. We used the following settings: AUC_0_ = 0.5, AUC_1_ = 0.80, power = 0.9, alpha = 0.05, allocation ratio = 1, lower false positive rate (FPR) = 0, upper FPR = 1.00, type of data = continuous, and alternative hypothesis = two-sided test, therefore the least number of measurements required was 34.

Continuous variables were expressed as medians (with interquartile ranges [IQR]) and compared between groups using the Wilcoxon or Friedman rank sum tests. For pairwise multiple comparisons, we used the Nemenyi post hoc test. Categorical variables were expressed as numbers (and percentages) and compared using the Fisher's exact test. Linear regression analysis was used to demonstrate relationships between percent change of VTI (ΔVTI) induced by the Trendelenburg position, and fluid challenge. Fluid responsiveness was defined as a VTI increase of 15% or more after volume expansion [17].

Receiver operating characteristic (ROC) curves were generated to evaluate percent changes in VTI and arterial pressure parameters (ΔSBP, ΔDBP and ΔPP), induced by the Trendelenburg maneuver to predicts fluid responsiveness. The area under ROC curves (AUROC) were compared using the DeLong test [25]. Sensitivity, specificity, positive and negative predictive values (PPV and NPV), and associated 95% confidence intervals (CI) were calculated based on the cutoff value as determined by the Youden Index (specificity + sensitivity − 1) [26]. To evaluate the variation of best threshold, we conducted a gray-zone analysis, as described by Georges et al*.* [17]. To visualize whether a predictive test can recognize the positive events fully and accurately, the recall (i.e., true positive rate)**-**precision (i.e., PPV) curves (PRC) were generated and area under precision–recall curve (AUPRC) were calculated [27].

All statistical tests were two-tailed, and a value of *p* < 0.05 indicated statistical significance. Statistical analyses were performed using R, version 3.6.2 (R Foundation for Statistical Computing, Vienna, Austria).

## Results

### Patients

The study flowchart is shown in Additional file 1: Figure S1. Due to unavailability of echocardiography staff in personal vacations, the study period became inconsecutive and 3 patients were not included. Besides, 3 (9%) patients were excluded for poor echogenicity. The main characteristics of the 22 enrolled patients are shown (Table 1). The major surgical procedures were heart transplantation (45%), valve surgery (32%), and Sun’s procedure [28] (14%). The median length of VA-ECMO support was 6 days (IQR 4–10), and 17 patients (77%) were weaned from VA-ECMO. IABP, RRT and iNO were performed in 1 (5%), 11 (50%) and 13 (59%) patients, respectively. The median length of MV was 16 days (IQR 7–23), and 14 patients (64%) underwent a tracheotomy. Eight patients (36%) died during a median length stay of 20 days (IQR 14–36) in ICU.

**Table 1:** Characteristics and clinical outcomes of included population

Variables	
Patients (*n*)	22
Demographic information	
Gender, *n* (%)	15 (68)
Age, year	57 [43–63]
BMI, kg/m^2^	24 [21–26]
Surgical procedure	
Heart transplantation, *n* (%)	10 (45)
Valve surgery, *n* (%)	7 (32)
Sun's procedure, *n* (%)	3 (14)
Others, *n* (%)	2 (9)
Postoperative critical ill status	
APACHE II score	20 [18–23]
LVEF, %	52 [36–62]
Concomitant therapies	
IABP, *n* (%)	1 (5)
RRT, *n* (%)	11 (50)
iNO, *n* (%)	13 (59)
Clinical outcomes	
Length of MV, day	16 [7–23]
Tracheotomy, *n* (%)	14 (64)
Length of ECMO, day	6 [4–10]
Weaning from ECMO, *n* (%)	17 (77)
Length of ICU stay, day	20 [14–36]
Length of hospital stay, day	36 [26–42]
Hospital mortality, *n* (%)	8(36)

Values are median [IQR] or number of patients (*n*)

*BMI* body mass index, *APACHE II score* Acute Physiology and Chronic Health Evaluation II score, *LVEF* left ventricular ejection fraction, *ECMO* extracorporeal membrane oxygenation, *IABP* intra-aortic balloon pump, *RRT* renal replacement therapy, *iNO* inspired nitric oxide, *MV* mechanical ventilation, *ICU* intensive care unit

### Measurements

Thirty-nine measurements were performed at a median of 4 days (IQR 3–5) after VA-ECMO support initiation (Table 2). Of these measurements, volume expansions were decided for hypotension, oliguria, skin mottling, and attempt to reduce vasopressor dose in 30 (77%), 4 (10%), 1 (3%) and 4 (10%) measurements, respectively. All laboratory examinations and supportive therapies are presented (Table 2). ECMO flow, tidal volume, and equivalent inotropic scores were 2.4 L/min (IQR 2.2–2.8), 7.0 mL/kg (IQR 6.7–7.6), and 6 μg/kg/min (IQR 1–12), respectively. During the study protocol, no patients developed cannula-related complications (i.e., hemorrhages, thrombosis or cannula displacement) following the Trendelenburg maneuver.

**Table 2:** Laboratory examinations and supportive therapies

Variables	
Measurements (*n*)	39
Laboratory and blood gas variables	
Bilirubin, μmol/L	28 [17–62]
Hemoglobin, g/L	84 [75–90]
Platelet, 10^9^/L	56 [33–71]
PaCO_2_, mmHg	36 [32–40]
PaO_2_, mmHg	101 [80–177]
Lactate, mmol/L	1.4 [1.2–1.7]
Ventilation setting	
FiO_2_, %	50 [50–55]
Tidal volume, mL/kg of PBW	7.0 [6.7–7.6]
PEEP, cmH_2_O	6 [5–8]
ECMO setting	
Blood flow, L/min	2.4 [2.2–2.8]
Blood flow, mL/kg/min	37 [31–43]
FdO_2_, %	60 [50–70]
Sweep gas flow, L/min	2.5 [2.4–3.0]
Pump rotation speed, round/min	2800 [2550–3150]
Implementation to measurement, day	4 [3–5]
Measurement to decannulation or death, day	2 [1–3]
Vasopressors and inotropes	
Norepinephrine, *n* (%)	19 (49)
Dose, µg/kg/min	0.11 [0.06–0.15]
Epinephrine, *n* (%)	14 (36)
Dose, µg/kg/min	0.03 [0.02–0.06]
Dobutamine, *n* (%)	20 (51)
Dose, µg/kg/min	1.1 [1.0–1.6]
Milrinone, *n* (%)	4 (10)
Dose, µg/kg/min	0.1 [0.1–0.2]
Equivalent inotropic score, µg/kg/min	6 [1–12]

Values are median [IQR] or number of patients (*n*)

*PaO*_*2*_ arterial partial pressure of oxygen, *PaCO*_*2*_ arterial partial pressure of carbon dioxide, *FiO*_*2*_ inspiratory fraction of oxygen, *FdO*_*2*_ oxygen concentration of device, *PEEP* positive end-expiratory pressure, *ECMO* extracorporeal membrane oxygenation, *PBW* predicted body weight

### The effects of the Trendelenburg maneuver and fluid challenge

**Fig. 2:**
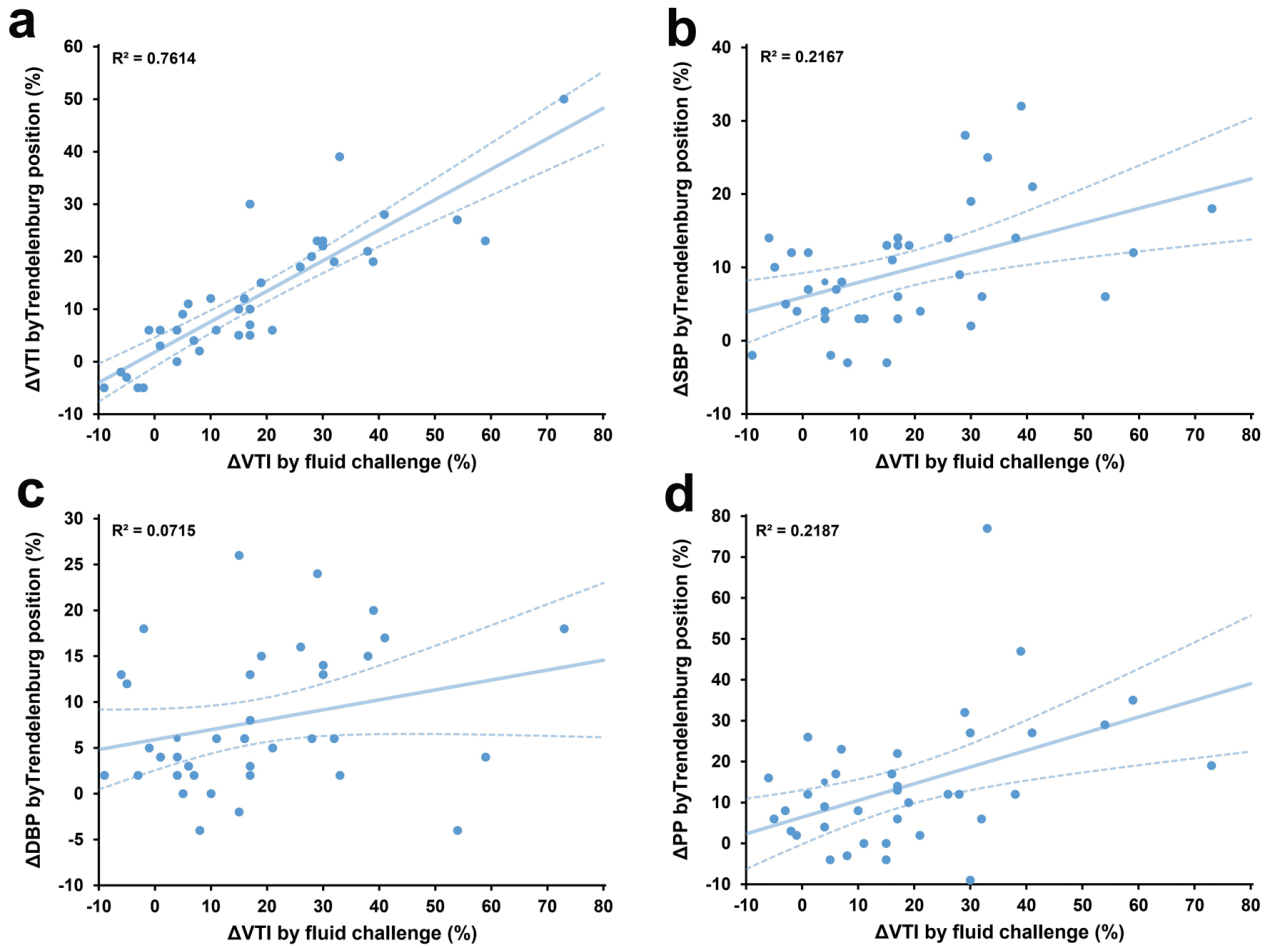
Linear regression between changes in velocity–time integral (ΔVTI, **a**), systolic blood pressure (ΔSBP, **b**), diastolic blood pressure (ΔDBP, **c**) and pulse pressure (ΔPP, **d**) induced by the Trendelenburg position and the fluid challenge. Solid and dashed lines indicate regression lines and their 95% confidential intervals

**Fig. 3:**
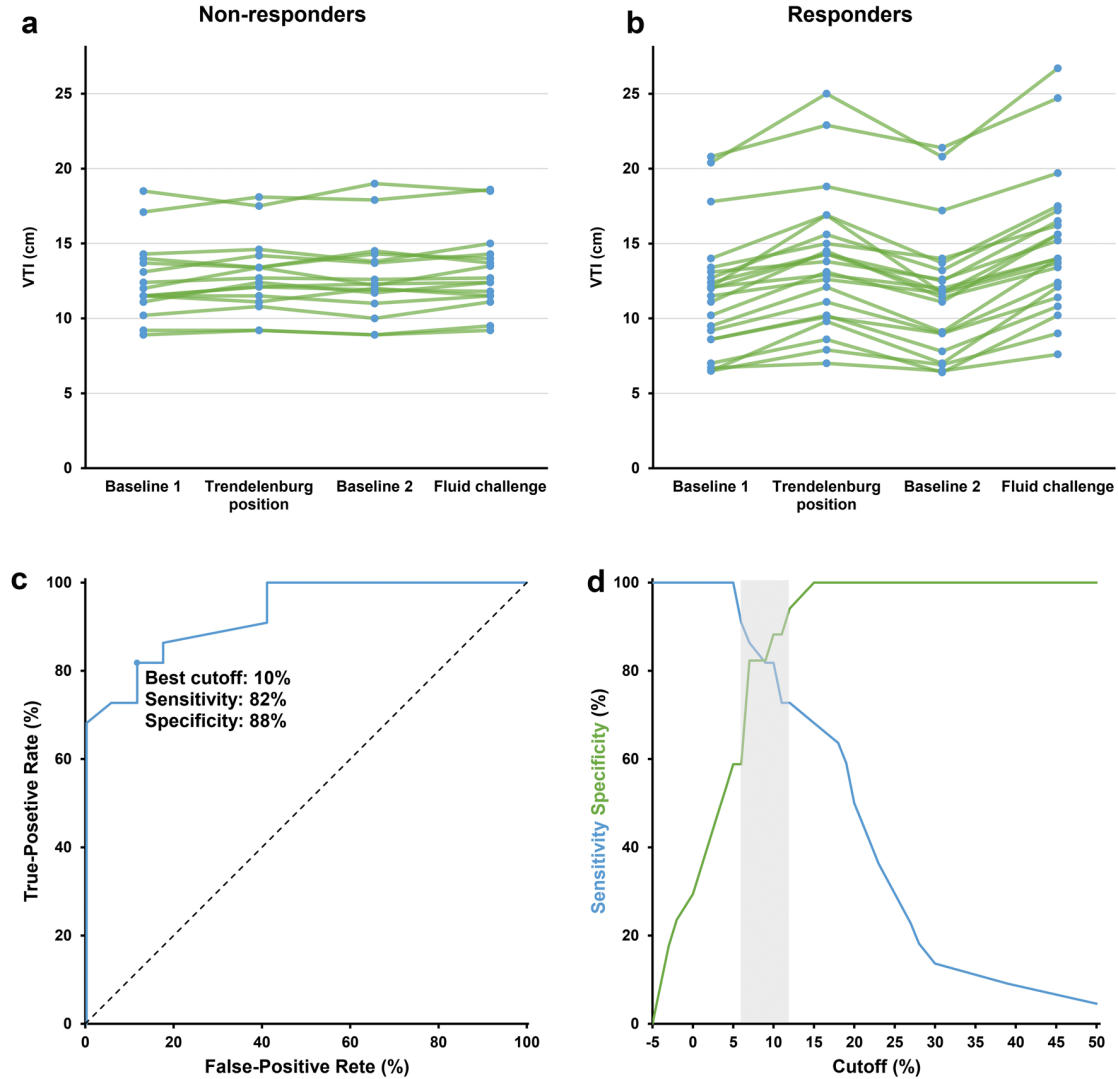
Individual values of velocity–time integral (VTI) of each step in non-responders (**a**) and responders (**b**) as well as receiver operating characteristics curve (**c**) and grey zone analysis (**d**) of changes in VTI induced by the Trendelenburg position to predict fluid responsiveness

Fluid responsiveness was observed in 22 of the 39 measurements (56%). Hemodynamic parameters from each step are shown (Table 3 and Fig. 3a, b). Higher baseline CVP was observed in non-responders. No variables recorded after return to baseline values exhibited significant differences. CVP, SBP, DBP, PP, and VTI indices were higher for either Trendelenburg or fluid challenges than their corresponding baseline values. The fluid challenge induced higher ΔSBP (9% [IQR 5–18%] vs. 8% [IQR 4–14%], *p* = 0.019), ΔPP (17% [IQR 1–21%] vs. 12% [IQR 4–21%], *p* = 0.044), and ΔVTI (16% [IQR 4–30%] vs. 10% [IQR 5–21%], *p* < 0.001) than the Trendelenburg maneuver. The ΔVTI between the Trendelenburg position and fluid challenge was highly related, with an *R*^2^ of 0.7614 and a slope of 0.58 (Fig. 2a). In comparison, the *R*^2^ of ΔSBP, ΔDBP and ΔPP were 0.2167, 0.0715 and 0.2187, respectively (Fig. 2b–d).

**Table 3:** Hemodynamic parameters at baselines, at the Trendelenburg position and after fluid challenge in responders (*n* = 22) and non-responders (*n* = 17)

Variables	Baseline 1	Trendelenburg position	Baseline 2	Fluid challenge	*p* value
HR, beat/min	105 [90–115]	107 [89–113]	106 [88–113]	106 [87–113]	0.232
Responders	103 [86–110]	102 [85–112]	104 [85–110]	101 [84–109]	0.051
Non-responders	112 [93–116]	113 [98–115]	111 [92–104]	111 [98–101]	0.629
CVP, mmHg	13 [11–15]	15 [13–17]^b^	13 [10–15]	15 [13–17]^b^	< 0.001
Responders	12 [10–15]	14 [11–16]^b^	12 [9–14]	14 [12–17]^b^	< 0.001
Non-responders	14 [13–15]	15 [14–17]^b^	14 [13–15]^a^	15 [14–17]^b^	< 0.001
SBP, mmHg	98 [86–107]	107 [101–115]^b^	98 [89–106]	111 [105–120]^b^	< 0.001
Responders	97 [85–109]	108 [102–115]^b^	98 [87–107]	116 [109–123]^b^	< 0.001
Non-responders	98 [87–105]	105 [99–111]^b^	98 [90–102]	105[94–111] ^a, b^	< 0.001
DBP, mmHg	59 [51–68]	63 [56–72]^b^	60 [53–68]	64 [58–72]^b^	< 0.001
Responders	56 [48–67]	62 [54–71]^b^	58 [48–68]	63 [60–72]^b^	< 0.001
Non-responders	60 [54–68]	63 [57–72]^b^	60 [55–68]	66 [57–71]^b^	< 0.001
PP, mmHg	37 [31–45]	44 [38–48]^b^	39 [31–45]	46 [38–55]^b^	< 0.001
Responders	41 [36–47]	46 [40–51]^b^	40 [32–47]	51 [46–57]^b^	< 0.001
Non-responders	36 [26–43]	38 [32–45]^a^	36 [30–42]	38 [30–45]^a^	0.035
VTI, cm	11.5 [9.4–13.3]	12.9 [11.0–14.6]^b^	11.9 [9.1–13.7]	13.7 [11.5–15.6]^b^	< 0.001
Responders	11.8 [8.8–13]	13.5 [10.4–15.5]^b^	11.7 [9.0–13.1]	14.0 [12.2–16.4]^b^	< 0.001
Non-responders	11.5 [11.1–13.7]	12.4 [11.1–13.4]	12.2 [11.0–13.8]	12.4 [11.5–14.0]	0.008

Values are median [IQR]

*HR* heart rate, *SBP* systolic blood pressure, *DBP* diastolic blood pressure, *CVP* central venous pressure, *PP* pulse pressure, *VTI* velocity–time integral

^a^*p* < 0.05, comparison between responders (*n* = 22) and non-responders (*n* = 17)

^b^*p* < 0.05, comparison between Trendelenburg position and baseline 1 or fluid challenge and baseline 2

### Prediction of fluid responsiveness

Data on fluid responsiveness predictions are shown (Fig. 3 C&D, Additional file 1: Figure S2 and Table 4). The AUROC of ΔVTI induced by Trendelenburg to predict fluid responsiveness was 0.93 (95% CI 0.81–0.98), with a sensitivity of 82% (95% CI 60–95%), and specificity of 88% (95% CI 64–99%), at a best threshold of 10%. The corresponding gray-zone was 6–12%, covering 32% of measurements. The percent change in arterial pressure variables, i.e., ΔSBP, ΔDBP and ΔPP, displayed lower predictive accuracies (AUROCs: 0.76 [95% CI 0.56–0.87], 0.73 [95% CI 0.52–0.85] and 0.69 [95% CI 0.48–0.82], respectively), whereas wider ranges of best thresholds (13% [95% CI 6–14%], 6% [95% CI 2–13%] and 10% [95% CI 2–23%]) than ΔVTI. Also, the ΔVTI showed higher AUPRC (0.96 [95% CI 0.88–0.99]) than ΔPP (0.70 [95% CI 0.61–0.92]), ΔSBP (0.85 [95% CI 0.71–0.95]) and ΔDBP (0.80 [95% CI 0.61–0.94]) (Additional file 1: Figure S3).

**Table 4:** Predictive parameters of receiver operating characteristic (ROC) curves of variable percent changes induced by Trendelenburg position

Parameters	ΔVTI	ΔPP	ΔSBP	ΔDBP
AUROC	0.93 (0.81–0.98)	0.69 (0.48–0.82)	0.76 (0.56–0.87)	0.73 (0.52–0.85)
Youden index	0.70	0.37	0.49	0.39
Best threshold	10%	10%	13%	6%
Gray zone	6–12%	2–23%	6–14%	2–13%
Values in gray zone	32%	62%	51%	62%
Sensitivity	82 (60–95)	73 (50–89)	55 (32–76)	68 (45–86)
Specificity	88 (64–99)	65 (38–86)	94 (71–100)	71 (44–90)
PPV	90 (68–99)	73 (50–89)	92 (64–100)	75 (51–91)
NPV	79 (54–94)	65 (38–86)	62 (41–80)	63 (38–84)

Values are median true value (95% confidence interval). Best threshold value was determined using the Youden index

The AUROC of ΔVTI was higher than ΔPP (*p* = 0.004), ΔSBP (*p* = 0.025) and ΔDBP (*p* = 0.018). There were no statistical differences among AUROCs of ΔPP, ΔSBP and ΔDBP (ΔPP vs. ΔSBP, *p* = 0.285; ΔPP vs. ΔDBP, *p* = 0.760; ΔSBP vs. ΔDBP, *p* = 0.645)

*AUROC* area under the receiver operating characteristic curve, *PPV* positive predictive value, *NPV* negative predictive value, *SBP* systolic blood pressure, *DBP* diastolic blood pressure, *PP* pulse pressure, *VTI* velocity–time integral, *Δ* value indicate percent change of each variable

## Discussion

To the best of our knowledge, this is the first study to explore the predictive value of ΔVTI, induced by the Trendelenburg position for fluid responsiveness in patients with VA-ECMO support. Our work demonstrated that ΔVTI monitoring during the Trendelenburg position was a reliable parameter in predicting fluid responsiveness in this population.

VA-ECMO could provide extracorporeal life support, as well as reducing the workload of the injured heart, thereby creating favorable conditions for myocardial recovery [1, 2]. During this support, clinicians usually increase ECMO flow to correct hypotension, which would lead to a higher left ventricular afterload and a downward shift of the Frank–Starling curve [29]. Apart from device flow, native cardiac output also plays an important role in maintaining systemic circulation, especially when the heart is in recovery, and device flow decreases correspondingly. Moreover, the volume status of the heart varies at any time due to hemorrhage, capillary leak, fluid therapy and changes in cardiac function, and hence should be dynamically evaluated.

Fluid responsiveness, as a cardiac response parameter to additional preload, is typically used for septic shock patients [8]; however, it may also be useful for patients with VA-ECMO support. On one hand, during VA-ECMO support, traditional parameters such as ejection fraction may not fully reflect cardiac function [30]. Fluid responsiveness may indicate whether the heart works at the steeper part of the Frank–Starling curve, thus guiding volume administration for hypotension, thereby providing indications for myocardial recovery and weaning feasibility from VA-ECMO. On the other hand, fluid unresponsiveness could also be used to guide fluid removal in ventilated patients with fluid overload [31], which may induce heart congestion, pulmonary edema, and acute kidney injury [32, 33], potentially increasing mortality [34]. Hence, assessment of fluid responsiveness could help to optimize preload and also evaluate cardiac function.

The Trendelenburg maneuver is a method that facilitates “autotransfusion”. Geerts et al. concluded that the final changes in cardiac output induced by the Trendelenburg position were similar to PLR [13]. In this study, the Trendelenburg position induced a 58% change in VTI, induced by a 500-mL fluid challenge, indicating a similar physiological effect to PLR. The high correlation between changes in VTI induced by the Trendelenburg position or fluid challenge allowed us to accurately predict fluid responsiveness in VA-ECMO patients.

Arterial pulsatility, coming from left ventricular ejection, is often considered as a marker of cardiac recovery during VA-ECMO [4, 35]. PP, as a parameter of pulsatile-flow, has been used as a predictor of successful weaning from VA-ECMO [36, 37]. However, in this study, changes in PP had a poorer predictive performance for fluid responsiveness than VTI. Although both variables could reflect the stroke volume, the PP is just a difference between SBP and DBP, rather than the area under the pulse contour per se. In fact, the relationship between SV and peripheral PP changes were not straightforward because they depended on arterial compliance, and pulse wave amplification from the aorta to the periphery. Previous studies have indicated that changes in PP exerted significant heterogeneity towards predictive accuracy for fluid responsiveness [38, 39]. Similarly, neither ΔSBP nor ΔDBP, induced by the Trendelenburg maneuver, demonstrated an acceptable predictive accuracy in this study.

### Study limitations

Our study had several limitations. First, it was conducted in a single center, which may have limited generalizability across different clinical settings. Second, a larger angle of Trendelenburg positioning may have introduced more “autotransfusion” to the central circulation [13], and was not evaluated in this study. Third, measurement of LVOT VTI is not the gold standard for evaluating cardiac output, because of its dependence on patient echogenicity and operator expertise. However, techniques based on thermodilution have proven unreliable in VA-ECMO patients [3], although they were accurate in estimating cardiac output under most circumstances [40]. Fourth, all patients in this study were under deep sedation, thus the Trendelenburg maneuver may be less reliable in predicting fluid responsiveness in non-sedated patients. Finally, because the study was conducted in a low VA-ECMO pump flow setting, our data may not be extrapolated to the acute phase of heart failure requiring full mechanical circulatory support.

## Conclusions

Our study suggested that an increase in VTI of at least 10%, induced by the Trendelenburg maneuver, was reliable in predicting fluid responsiveness in patients with VA-ECMO.

## Supplementary Information

**Additional file 1:** **Figure S1.** Study flowchart. **Figure S2.** Receiver operating characteristics curves and grey zone analyses. **Figure S3.** The precision–recall curves for changes in VTI, PP, SBP and DBP induced by Trendelenburg maneuver.

## Data Availability

All data generated or analyzed during this study are included in this published article.

## Abbreviations

VA-ECMO: Veno-arterial extracorporeal membrane oxygenation
SVV: Stroke volume variation
PPV: Pulse pressure variation
ΔIVCD: Respiratory variations in the inferior vena cava diameter
PLR: Passive leg raising
TTE: Transthoracic echocardiography
VTI: Velocity–time integral
ICU: Intensive care unit
HR: Heart rate
SBP: Systolic blood pressure
DBP: Diastolic blood pressure
CVP: Central venous pressure
PP: Pulse pressure
CV: Coefficient of variation
LSC: Least significant change
APACHE: Acute Physiologic and Chronic Health Evaluation
LVEF: Left ventricular ejection fraction
MV: Mechanical ventilation
RRT: Renal replacement therapy
iNO: Inspired nitric oxide
ROC: Receiver operating characteristic curve
PPV: Positive predictive values
NPV: Negative predictive values
CI: Confidence intervals
PRC: Precision–recall curves
