# Supplementary material for: Trendelenburg maneuver predicts fluid responsiveness in patients on veno-arterial extracorporeal membrane oxygenation
Source: Ann Intensive Care. 2021 Jan 26;11:16. doi: 10.1186/s13613-021-00811-x (PMC7838230; doi:10.1186/s13613-021-00811-x)
Supplement: Supplementary file 1 — Additional file 1: Figure S1. Study flowchart. Figure S2. Receiver operating characteristics curves and grey zone analyses. Figure S3. The precision–recall curves for changes in VTI, PP, SBP and DBP induced by Trendelenburg maneuver. [file 13613_2021_811_MOESM1_ESM.docx]

Content

Figure. S1. Study flowchart

Figure. S2. Receiver operating characteristics curves and grey zone analyses

Figure. S3. The precision-recall curves for changes in VTI, PP, SBP and DBP induced by Trendelenburg maneuver.

**22** patients enrolled in this study

**40** patients with VA-ECMO support

**10** patients were excluded

**3** echocardiography staff unavailable

Oct 1-14, 2018, May 1-12 and Oct 1-13, 2019

**3** unsatisfactory cardiac echogenicity

**2** pulselessness (pulse pressure<15mmHg)

**2** evidence of hypovolemia

1 persistent hemorrhage

1 drainage cannula fluttering

**32** patients eligible

**Figure S1**. Study flowchart


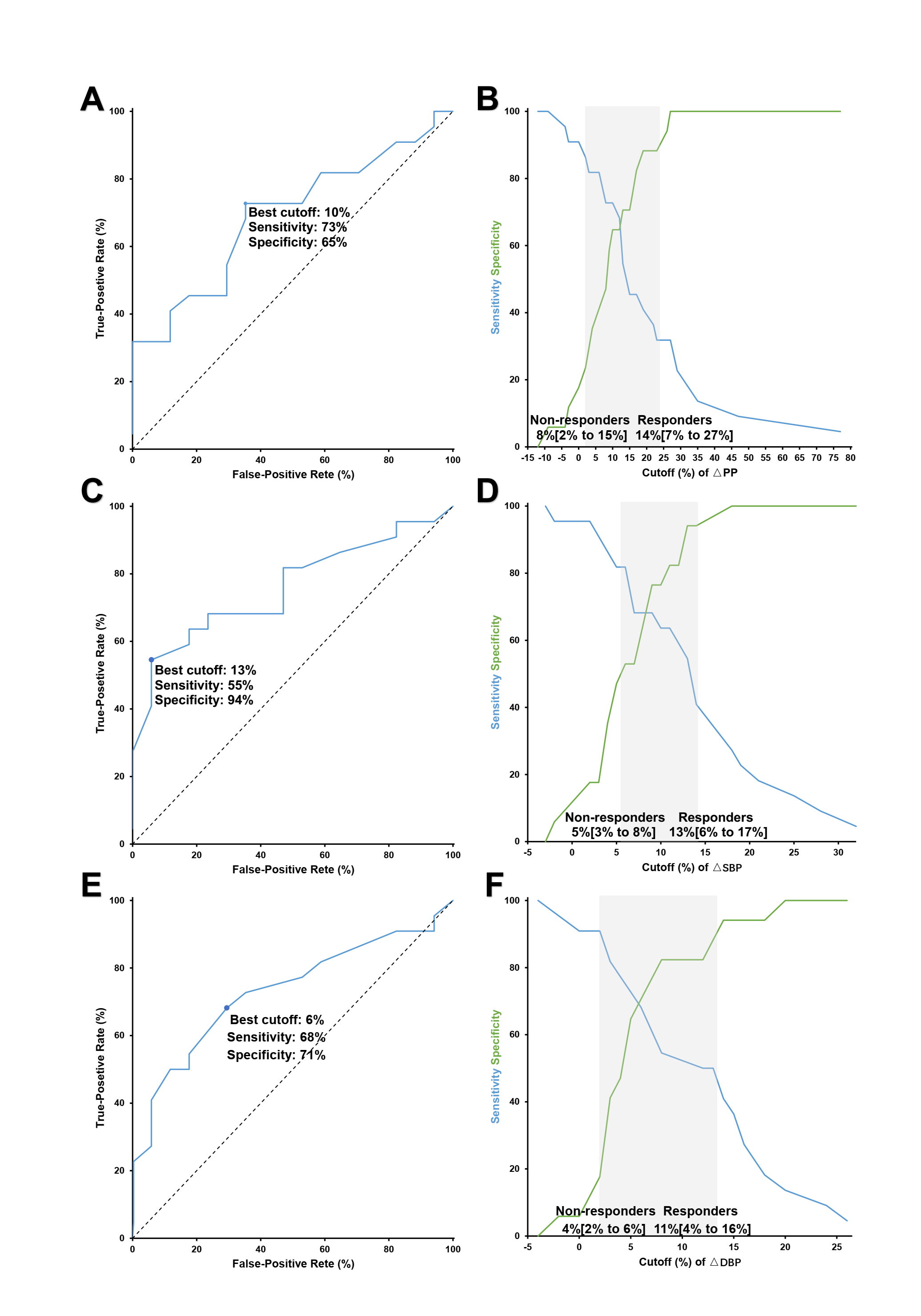


**Figure S2**. Receiver operating characteristics curves and grey zone analyses for Trendelenburg position induced changes in pulse pressure (**A&B**) systolic blood pressure (**C&D**) and diastolic blood pressure (**E&F**).


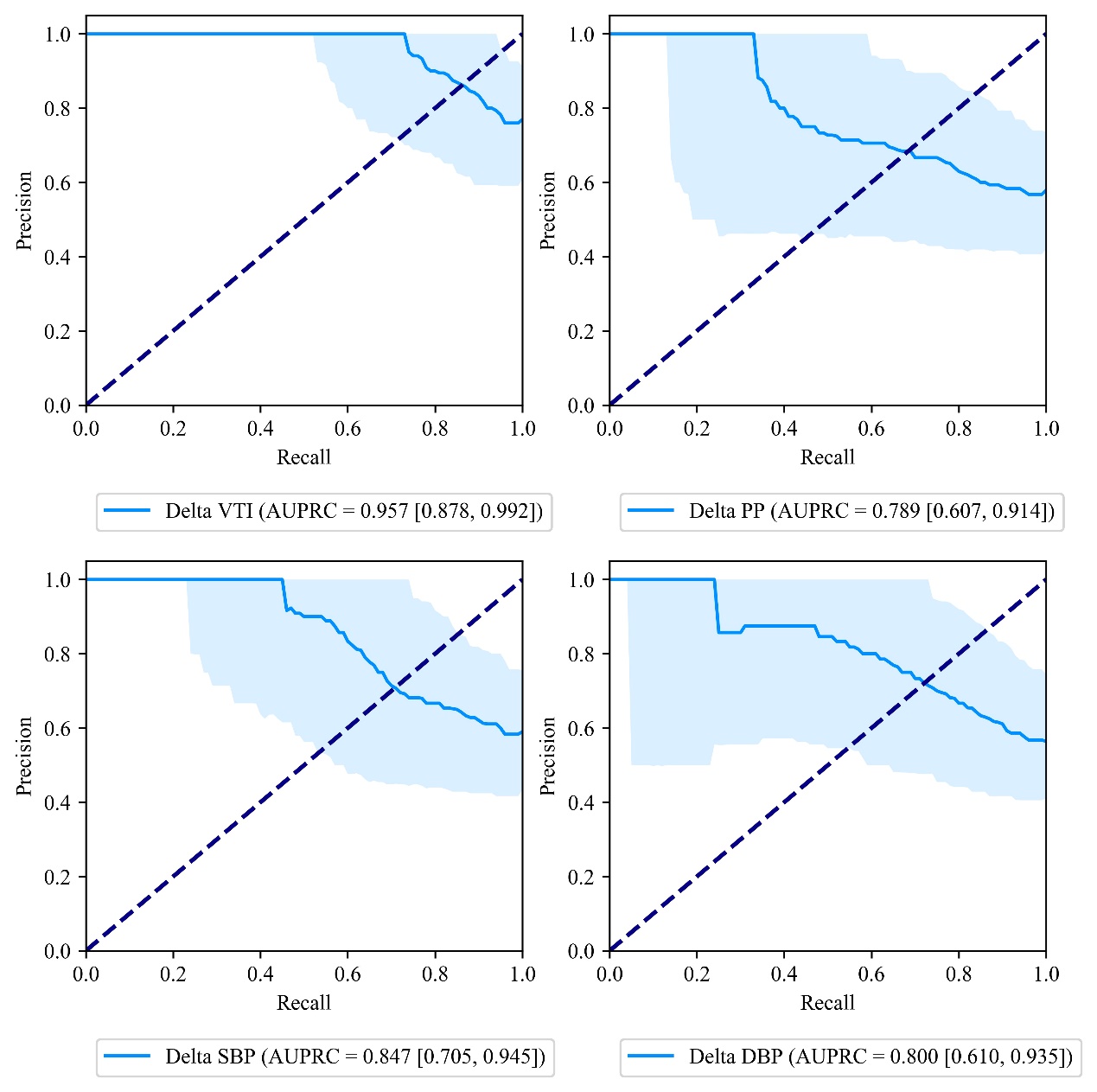


**Figure S3**. The precision-recall curves (PRC) for changes in VTI, PP, SBP and DBP induced by Trendelenburg maneuver. The blue areas indicate the 95% confidence interval. SBP systolic blood pressure, DBP diastolic blood pressure, PP pulse pressure, VTI velocity time integral, APRUC, the area under the PRC. The delta (Δ) value indicates percent change of each variable.
